# Correction: The novel SH3 domain protein Dlish/CG10933 mediates fat signaling in Drosophila by binding and regulating Dachs

**DOI:** 10.7554/eLife.22672

**Published:** 2016-11-08

**Authors:** Yifei Zhang, Xing Wang, Hitoshi Matakatsu, Richard Fehon, Seth S Blair

Zhang Y, Wang X, Matakatsu H, Fehon R, Blair SS. 2016. The novel SH3 domain protein Dlish/CG10933 mediates fat signaling in Drosophila by binding and regulating Dachs. *eLife* **5**:e16624. doi: 10.7554/eLife.16624.

Published 3, October 2016

We discovered an error in Figure 2F. As the original confocal images were being changed into the green-magenta color scheme, the magenta (Dachs) channel from the precursor for Figure 2E was inadvertently copied into the upper color image in 2F, and rendered into the lower black and white single channel image. We have replaced Figure 2F with the correct image. Correcting this error does not affect the finding that posterior expression of Fat△ECD△5-6 could also weakly reduce Dachs levels in a *fat* mutant wing disc.

The corrected Figure 2 is shown here:

**Figure 1:**
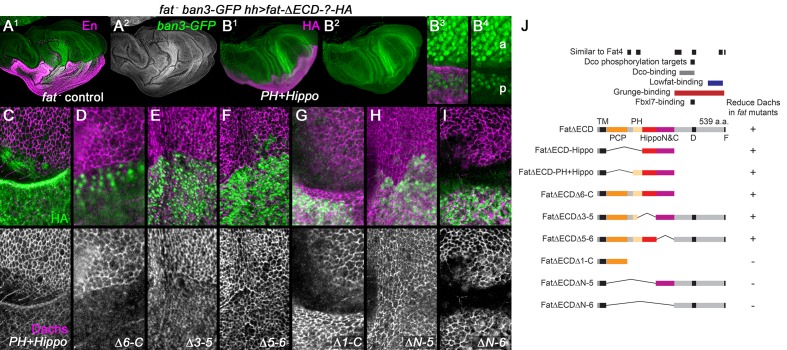

The originally published Figure 2 is also shown for reference:

**Figure 2:**
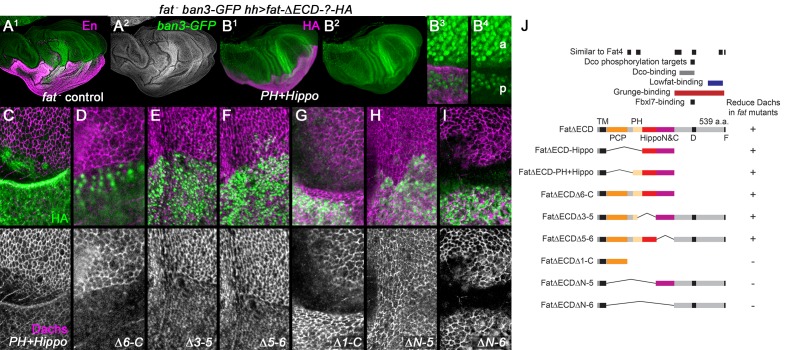

The article has been corrected accordingly.

