# Supplementary material for: The novel SH3 domain protein Dlish/CG10933 mediates fat signaling in Drosophila by binding and regulating Dachs
Source: eLife. 2016 Oct 3;5:e16624. doi: 10.7554/eLife.16624 (PMC5047748; doi:10.7554/eLife.16624)
Supplement: Figure 7—source data 1. — DOI: http://dx.doi.org/10.7554/eLife.16624.016 [file elife-16624-fig7-data1.docx]

| Wing area (normalized to *nub-gal4* average) | *nub-gal4* | *nub-gal4 UAS-dlish-RNAi* |
| --- | --- | --- |
|  | 0.970 | 0.895 |
|  | 0.966 | 0.912 |
|  | 0.986 | 0.898 |
|  | 0.958 | 0.843 |
|  | 1.120 | 0.848 |
|  |  | 0.804 |
|  |  | 0.812 |
|  |  | 0.768 |
|  |  | 0.853 |
|  |  | 0.851 |
|  |  | 0.902 |
|  |  | 0.855 |
|  |  | 0.913 |
|  |  | 0.846 |
|  |  | 0.839 |
| **Average** | **1.000** | **0.856** |
| Standard Deviation | 0.068 | 0.042 |
| p= (single-tailed T test) |  | 0.00321 |
| p= (single-tailed Whitney-Mann test) | | 0.00062 |
