# Supplementary material for: The novel SH3 domain protein Dlish/CG10933 mediates fat signaling in Drosophila by binding and regulating Dachs
Source: eLife. 2016 Oct 3;5:e16624. doi: 10.7554/eLife.16624 (PMC5047748; doi:10.7554/eLife.16624)
Supplement: Figure 10—source data 1. — Tests shown are two-tailed. The slight increase in dlish04 fatfd double mutants in comparison to dlish04 did not reach statistical significance, and was significantly lower that the added average increases of dlish04 and fatfd (Wilcoxon Rank Sum test or single sample T test to medians or means of 6.86). The N for dlish04 fatfd is too low to obtain an exact p value using the Wilcoxon test, but still predicts a significance cutoff. DOI: http://dx.doi.org/10.7554/eLife.16624.021 [file elife-16624-fig10-data1.docx]

|  |  | **Dachs/Tubulin** | | | |  |
| --- | --- | --- | --- | --- | --- | --- |
|  |  | Fold change compared to wild type | | | | |
|  |  | *dlish^04^* | *fat^fd^* | | *dlish^04^ fat^fd^* | |
|  |  | 2.12 |  | | 2.24 | |
|  |  | 1.53 |  | | 2.61 | |
|  |  | 3.41 |  | | 4.32 | |
|  |  | 2.60 | 3.43 | | 3.61 | |
|  |  | 2.51 | 3.54 | | 3.49 | |
|  |  | 3.81 | 4.61 | | 5.05 | |
|  |  | 3.81 | 4.20 | | 4.64 | |
|  |  | 3.32 | 4.07 | | 3.83 | |
|  | **Average** | **2.89** | **3.97** | | **3.72** | |
|  | SD | 0.83 | 0.49 | | 0.96 | |
| Whitney-Mann to *dlish^04^* | |  | |  | | p=0.066 |
| T test to *dlish^04^* | |  | |  | | p=0.084 |
| Wilcoxon to 6.86 (*dlish^04^* + *fat^fd^* averages) | | | |  | | p≤ 0.05 |
| T test to 6.86 (*dlish^04^ + fat^fd^* averages) | | | |  | | p=0.000036 |
