# Supplementary material for: The novel SH3 domain protein Dlish/CG10933 mediates fat signaling in Drosophila by binding and regulating Dachs
Source: eLife. 2016 Oct 3;5:e16624. doi: 10.7554/eLife.16624 (PMC5047748; doi:10.7554/eLife.16624)
Supplement: Figure 11—source data 1. — DOI: http://dx.doi.org/10.7554/eLife.16624.024 [file elife-16624-fig11-data1.docx]

|  | posterior area / anterior area | | |  |
| --- | --- | --- | --- | --- |
|  | *hh-gal4* | *hh>app* | *hh>dlish* | *hh>app+dlish* |
|  | 0.99 | 0.87 | 1.04 | 1.22 |
|  | 1.01 | 0.84 | 1.06 | 1.18 |
|  | 1.00 | 0.96 | 1.09 | 1.25 |
|  | 1.02 | 0.89 | 1.13 | 1.26 |
|  | 1.00 | 0.85 | 1.14 | 1.21 |
| **Average** | **1.00** | **0.88** | **1.09** | **1.22** |
| Standard deviation | 0.01 | 0.05 | 0.04 | 0.03 |
|  | *hh-gal4* vs *hh>app* | | *hh>dlish* vs *hh>app+dlish* | |
| p= (single-tailed T test) |  | 0.0016 |  | 0.00045 |
| p= (single-tailed Whitney-Mann test) | | 0.00604 |  | 0.00604 |
