# Supplementary material for: The novel SH3 domain protein Dlish/CG10933 mediates fat signaling in Drosophila by binding and regulating Dachs
Source: eLife. 2016 Oct 3;5:e16624. doi: 10.7554/eLife.16624 (PMC5047748; doi:10.7554/eLife.16624)
Supplement: Figure 12—source data 1. — Strength of streptavidin binding and anti-FLAG binding was quantified from western blots. To adjust for differences between each trial’s overall staining levels, the experimental streptavidin/anti-FLAG numbers were normalized to the control condition for the same trial, and the number expressed as a fold change. Comparisons were to a hypothetical median of 1 using the two-tailed Wilcoxon Rank Sum test, and to a hypothetical mean of 1 using a two-tailed single-sample T test. The N for App + FatΔECD / App alone is too low to obtain an exact p value using the Wilcoxon test, but still predicts a significance cutoff. DOI: http://dx.doi.org/10.7554/eLife.16624.026 [file elife-16624-fig12-data1.docx]

| Change in proportion of biotinylated Dlish  (Streptavidin / anti-FLAG) | | | |
| --- | --- | --- | --- |
| Fold Change | App /  no App |  | App + FtΔECD / App alone |
|  | 3.17 |  | 0.43 |
|  | 1.44 |  | 0.12 |
|  | 2.09 |  | 0.29 |
|  | 3.44 |  | 0.30 |
|  | 2.52 |  | 0.16 |
|  | 6.87 |  | 0.42 |
|  | 3.39 |  |  |
|  | 0.74 |  |  |
|  | 1.17 |  |  |
|  | 2.00 |  |  |
|  | 1.18 |  |  |
|  | 2.04 |  |  |
|  | 2.02 |  |  |
|  | 2.24 |  |  |
| Average | **2.45** |  | **0.29** |
| Standard deviation | 1.52 |  | 0.13 |
| p = (two-tailed Wilcoxon   test) | 0.0019 |  | ≤ 0.05 |
| p = (two-tailed T test) | 0.0034 |  | 0.00004 |
